# Proper development of long-lived memory CD4 T cells requires HLA-DO function

**DOI:** 10.3389/fimmu.2023.1277609

**Published:** 2023-10-16

**Authors:** Nianbin Song, Robin A. Welsh, Scheherazade Sadegh-Nasseri

**Affiliations:** Department of Pathology, Johns Hopkins University, School of Medicine, Baltimore, MD, United States

**Keywords:** HLA-DO, CD4 memory cell, memory T cell development, memory T cell maintenance, antigen processing and presentation

## Abstract

**Introduction:**

HLA-DO (DO) is an accessory protein that binds DM for trafficking to MIIC and has peptide editing functions. DO is mainly expressed in thymic medulla and B cells. Using biochemical experiments, our lab has discovered that DO has differential effects on editing peptides of different sequences: DO increases binding of DM-resistant peptides and reduces the binding of DM-sensitive peptides to the HLA-DR1 molecules. In a separate line of work, we have established that appropriate densities of antigen presentation by B cells during the contraction phase of an infection, induces quiescence in antigen experienced CD4 T cells, as they differentiate into memory T cells. This quiescence phenotype helps memory CD4 T cell survival and promotes effective memory responses to secondary Ag challenge.

**Methods:**

Based on our mechanistic understanding of DO function, it would be expected that if the immunodominant epitope of antigen is DM-resistant, presentation of decreased densities of pMHCII by B cells would lead to faulty development of memory CD4 T cells in the absence of DO. We explored the effects of DO on development of memory CD4 T cells and B cells utilizing two model antigens, H5N1-Flu Ag bearing DM-resistant, and OVA protein, which has a DM-sensitive immunodominant epitope and four mouse strains including two DO-deficient Tg mice. Using Tetramers and multiple antibodies against markers of memory CD4 T cells and B cells, we tracked memory development.

**Results:**

We found that immunized DR1^+^DO-KO mice had fewer CD4 memory T cells and memory B cells as compared to the DR1^+^DO-WT counterpart and had compromised recall responses. Conversely, OVA specific memory responses elicited in HA immunized DR1+DO-KO mice were normal.

**Conclusion:**

These results demonstrate that in the absence of DO, the presentation of cognate foreign antigens in the DO-KO mice is altered and can impact the proper development of memory cells. These findings provide new insights on vaccination design leading to better immune memory responses.

## 1 Introduction

The proper development and homeostasis of memory CD4 T cells have been known as key processes in determining good quality immunological memory responses. Many studies have been devoted to understanding factors contributing to this process from various perspectives including regulation of germinal center reaction (GC reaction), TCR signaling strength, and regulation of glucose/lipid metabolism (1–5). However, little effort has been given to investigating how the quantity of presented peptide/MHC complexes impacts the development of memory T cells.

HLA-DO (DO or H2-O in mice) is a non-classical MHC Class II accessory molecule that is expressed selectively in thymic medulla, B cells (6–8) and some subsets of immature dendritic cells (DC) (9–11). Despite this selective expression pattern, the function of DO has remained elusive as the knockout mice do not spontaneously develop any disease phenotype despite minor indications of autoimmunity (12). One important clue pointing to the function of DO comes from its physical association with HLA-DM (DM, or H-2M in mice), the main MHCII peptide editor. This association has been found to be necessary for DO to be exported out of ER (13). DM is required for removing Class II Invariant Chain peptide (CLIP) from the newly synthesized MHC II molecule and generating a peptide-receptive conformation of MHC II, so that an MHC II groove fitting epitopes can bind (14). Thus, many studies have linked the function of DO with DM, proposing DO could bind and inhibit DM by blocking the access of DM to MHC II molecules (15–19). However, our more recent studies have found that DO could play a fine-tuning role in this peptide selection process by stabilizing MHC II peptide-receptive conformation generated by removal of CLIP or other poorly fitting peptides by DM. As a result, the binding of a good MHC groove fitting peptide that is not prone to be removed by DM (or known as DM-resistant peptide) to MHCII will be enhanced in the presence of DO (20, 21).

We and others have described altered self-antigen presentation in I-Ab^+^H2-O KO and DR1+H2-O KO mice that leads to altered CD4 TCR repertoires and susceptibility to autoimmunity (12, 22). Nevertheless, as the proposed impact of DO on antigen (Ag) presentation is based on the DM sensitivity of each epitope rather than the source of epitopes, it is reasonable to expect that DO deficiency will not only alter presentation of self-peptides, but also impacts foreign epitope presentation, which could result in altered immune responses against foreign antigens. However, despite findings from several human GWAS studies linking DO gene with HCV infection and cancer (23–28), most studies up to date on DO have been focusing only on its association with autoimmunity in mice and much less concern has been given on the role of DO in infectious diseases except one study that linked loss of DO beta gene to enhanced production of broadly neutralizing antibodies against a viral infection (25).

Previously, our lab has found that CD4 memory T cells become quiescent during the contraction phase of infections, a necessary requirement for their long-term survival. B cell presentation of low levels of cognate antigenic peptides bound to MHCII proved essential for development of quiescence state (29–33). Based on these findings, together with the proposed mechanism for DO function, we hypothesized that presentation of reduced densities of cognate antigens in H2-O KO mice may disrupt the proper development of resting CD4 memory T cells.

Here, we utilized the DM-resistant, DR1-restricted, immunodominant epitope of H5N1-Flu vaccine (22, 34–38), and tracked specific memory development in DR1+H2-O WT and DR1+H2-O KO mice. We present data showing that fewer numbers of CD4 memory T cells and memory B cells were developed and responded poorly to a secondary challenge in H5N1-Flu vaccine immunized DR1+H2-O KO. Conversely, tracking development of memory CD4 T cells specific to OVA, which has a DM-sensitive immunodominant epitope (39), was unchanged. These results suggest that DO promotes development of long-lived memory CD4 T cells when the immunodominant epitope is DM-resistant.

## 2 Materials and methods

### 2.1 Mice

C57BL/6 (B6) mice were purchased from the Jackson Laboratory. I-Ab+H2-O KO mice used in the above experiments were generated by backcrossing 129.H2-O KO mice (Lars Karlsson, Johnson and Johnson Pharmaceutical Research and Development, San Diego, CA) onto B6 for 10 generations by P. Jensen and X. Chen (University of Utah) and kindly gifted to us. The original HLA-DR1 (DRB1*0101) (DR1) transgenic mice (obtained from Dr. Dennis Zaller, Merck) express a fusion MHC II molecule containing the DR1 binding groove and the membrane proximal domain of murine I–E molecule (40). The resulting DR1 mice were backcrossed to MHC class II KO mice for 12–16 generations to eliminate endogenous class II proteins (I-A^f^) and were then inbred to homozygosity. DR1+ H2-O KO mice were generated by crossing the I-Ab+ H2-O KO mice with transgenic DR1 mice for >10 generations to achieve H2-O KO homozygosity. The DR1+ H2-O WT were generated by crossing the DR1 mice with B6 mice for >10 generations. All DR1+H2-O WT and DR1+H2-O KO mice still express murine I-A^b^ molecules from the B6 background. All mice were housed in the Johns Hopkins University animal facilities under virus-free conditions. All experiments were performed in accordance with protocols approved by the Animal Care and Use Committee of the Johns Hopkins University School of Medicine.

### 2.2 Peptide, protein, H5N1 influenza vaccine and antibodies

The H5N1 HA (259–274) (SNGNFIAPEYAYKIVK) and OVA (326–339) (AVHAAHAEINEAGR) were synthesized by [Peptide 2.0] with >95% purity. The inactivated influenza vaccine, A/H5N1 Influenza Vaccine, was obtained from [beiresources.org]. The HA protein was obtained from [eEnzyme]. Percp-Cy55-CD19(6D5), Brilliant Violet 421-CD44(IM7), Brilliant Violet 421-CXCR5(L138D7), Alexa Fluor 700-CD4(GK1.5), Alexa Fluor 700-IgD(11-26c.2a), FITC-CD80(16-10A1), PE-PD-L2(TY25),PE-GL7(GL7), PE-Cy7-IgM(RMM-1), PE-Cy7-CD86(GL-1), PE-Cy7-CD69(H1.2F3), PE-Cy7-PD-1(29F.1A12), PE-Cy7-CD28(37.51), APC-CD95(SA367H8), APC-CD40(3/23), APC-B220(RA3-6B2), APC-CD11c(N418) and APC-F4/80(BM8) were from [Biolegend]. Brilliant Ultra Violet 395-CD3 (17A2) and Brilliant Ultra Violet 496-CD4 (RM4-4) were from [BD Biosciences]. Fixable viability dye eFluor 780 was from [eBioscience]. Celltrace CFSE proliferation dye and Celltrace violet dye were from [Thermo Fisher Scientific]. PE-CD99 (polyclonal) was from [R&D systems].

### 2.3 DR1/HA (259–274) tetramer and I-A^b^/OVA (329–337) tetramer

PE-conjugated DR1/H5N1-HA (259–274) tetramers were produced in our laboratory. Biotinylated DR1 monomers, PE-conjugated CLIP tetramers, and the conjugation protocol were provided by the NIAID Tetramer Core Facility. Steptavidin-PE was from [Thermo Fisher Scientific]. PE-conjugated I-A^b^/OVA (329–337) tetramer was provided by the NIAID Tetramer Core Facility.

### 2.4 Staining for MHC II tetramers

MHC class II tetramer staining was done as previously (40). Briefly, spleens of flu vaccine or OVA protein immunized H2-O WT or KO mice were harvested. Spleen cells were stained with PE-conjugated H5N1 HA tetramers or PE-conjugated OVA tetramers for 2 hours at 37°C in RPMI + 2% FBS + 0.1% azide in the presence of 50 nM Dasatinib [Cell Signaling Technology, Danvers, MA]. After staining for 2 hours, cells were washed with PBS before staining with antibodies for flow cytometry. For flow cytometry, the FACSymphony flow cytometer [BD Bioscience] was used for experiments in this study. FlowJo v10 software was used for data analysis.

### 2.5 Immunization of mice with H5N1 flu vaccine/H5N1 HA protein and OVA protein

6-8 weeks old DR1+H2-O KO or DR1+H2-O WT mice were immunized intraperitoneally with 9µg H5N1 vaccine + 50µg CpG (for memory development) or 9µg of HA protein + 50µg CpG (for primary response). Spleens were harvested either 7-10 days post immunization with HA protein for *ex vivo* staining of primary response or 3-4 months post immunization for *ex vivo* staining of memory CD4 T cells or *in vitro* re-stimulation. Immunization of I-A^b^+H2-O WT and I-A^b^+H2-O KO mice with OVA was done using same immunization strategy with 200µg of OVA protein and 50µg CpG for memory development or primary response.

### 2.6 *In vitro* re-stimulation of CD4 T cells from H5N1-Flu vaccine or OVA protein immunized mice

DR1+H2-O KO or DR1+H2-O WT mice were immunized with flu vaccine as described above. The spleen cells from the immunized mice were harvested 3-4 months post immunization and were labeled with CFSE proliferation dye before culturing with the presence or absence of peptide and IL-2. The cells were then harvested from culture for staining with antibodies for flow cytometry on day 6 post culture. Re-stimulation of CD4 T cells from OVA immunized I-A^b^+H2-O WT and I-A^b^+H2-O KO mice was done in the same way.

### 2.7 *In vivo* challenge of H5N1 flu vaccine or OVA immunized mice

DR1+H2-O KO or DR1+H2-O WT mice were immunized with flu vaccine as described above. The immunized mice were challenged with same dose of H5N1 vaccine and CpG at 4-6 months post immunization. The spleens and serum from the immunized mice were harvested 10 days post the challenge. The spleen cells were stained with antibodies for flow cytometry and the serum was used for antibody ELISA. The *in vivo* challenge of OVA immunized I-A^b^+H2-O WT and I-A^b^+H2-O KO mice and harvest of spleen/serum was performed in the same way.

### 2.8 Serum antibody ELISA

Sera from blood of flu vaccine or OVA immunized mice were isolated by clotting for 30 min and spinning down at 4°C. The 96-well flat-bottom high binding ELISA plate was coated with HA protein or OVA protein diluted in ELISA binding buffer [Bio-Rad] overnight at room temperature. The plate was washed and blocked with 5% Goat serum in TBS/tween-20. The 1:200 diluted serum samples were then added and incubated for 2 hours at room temperature. The 1:2000 diluted HRP conjugated goat anti-mouse IgG1, IgG2b, IgG2c, IgG3 and IgM [Cell Signaling Technology] were added to plate and incubated for 1 hour before adding TMB substrate [Thermofisher Scientific] and stop solution. The ELISA plate was then measured with a spectrophotometer at 450nm.

### 2.9 Statistical analyses

GraphPad Prism was used for all statistical analyses. A standard Student T-test was used for estimation of statistical significance. Data is shown as mean ± SEM. *p<0.05, **p<0.01, ***p<0.001, ****p<0.0001. Each experiment including 4 mice per group was independently repeated 3-4 times.

## 3 Results

### 3.1 Absence of H-2O impairs memory development in H5N1-Flu vaccine immunized mice

**Figure 1:**
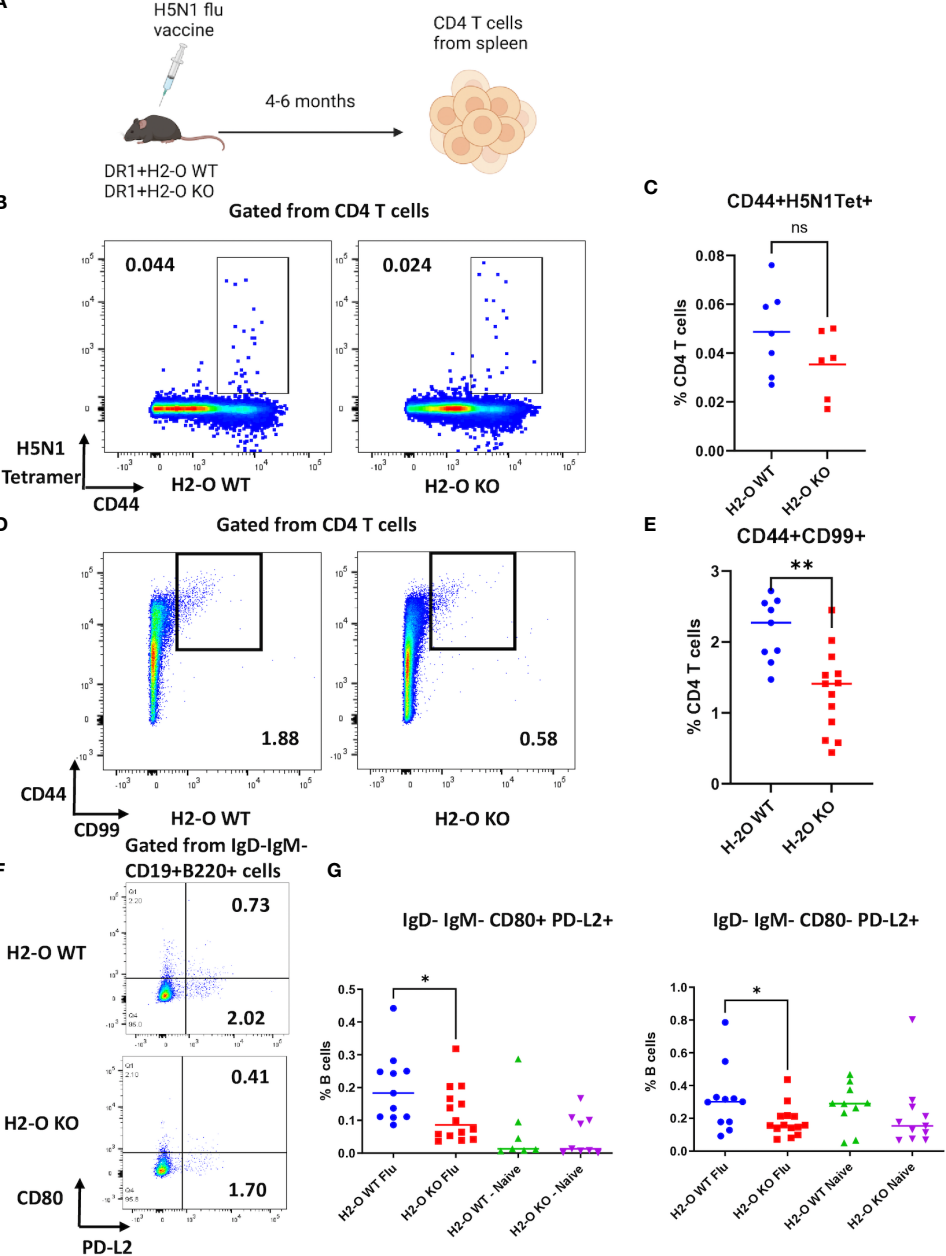
The absence of H2-O impairs memory development in flu vaccine immunized mice. **(A)** 6-8 weeks old DR1+H2-O WT mice and DR1+H2-O KO mice were intraperitoneally immunized with 9µg of inactivated H5N1 Influenza Vaccine and 50µg of CpG and sacrificed for spleen cells 4-6 months post immunization. The splenocytes were then divided for staining with either CD3, CD4, CD44 and CD99 antibodies (T cell panel) or B220, CD19, IgD, IgM, CD80 and PD-L2 antibodies (B cell panel) for flow cytometry. Each experiment including 4 mice per group was independently repeated 3 times. **(B)** Representative pseudocolor plots of CD44+H5N1 tetramer+CD4 T cells gated from live CD4 T cells in immunized DR1+H2-O WT mice (Left) and DR1+H2-O KO mice (Right). **(C)** Percentages of CD44+ H5N1 Tetramer+ CD4 T cells from spleens of immunized DR1+H2-O WT mice (blue) and DR1+H2-O KO mice (red). Each dot represents one individual mouse. P-value= 0.1634(two-tailed unpaired T-test). The experiment has been repeated 3 times. **(D)** Representative pseudocolor plots of CD44+CD99+ CD4 T cells in immunized DR1+H2-O WT mice (Left) and DR1+H2-O KO mice (Right). **(E)** Percentages of CD44+CD99+ CD4 T cells from spleens of immunized DR1+H2-O WT mice (blue) and DR1+H2-O KO mice (red). Each dot represents one individual mouse. P-value=0.0015 (two-tailed unpaired T-test). The experiment has been repeated 3 times. **(F)** Representative pseudocolor plots of IgD-IgM-CD19+B220+ spleen cells in immunized DR1+H2-O WT mice (Top) and DR1+H2-O KO mice (bottom). **(G)** Percentages of IgD-IgM-CD80+PD-L2+ (Left panel) and IgD-IgM-CD80-PD-L2+ (Right panel) memory B cell subsets in total CD19+B220+ B cells from immunized DR1+H2-O WT mice (Blue) and DR1+H2-O KO mice (Red). Same subsets of memory B cells from age-matched naïve DR1+H2-O WT mice (Green) and DR1+H2-O KO mice (Purple) were used as controls. The p-value for IgD-IgM-CD80+PD-L2+ cell subset between immunized DR1+H2-O WT and DR1+H2-O KO mice is 0.0395 (two-tailed unpaired T-test), the p-value for IgD-IgM-CD80-PD-L2+cell subset between immunized DR1+H2-O WT and DR1+H2-O KO mice is 0.0356 (two-tailed unpaired T-test). Each dot represents one individual mouse. Each experiment including 4 mice per group was independently repeated 4 times. Data is shown as mean ± SEM. *p<0.05, **p<0.01, ns, not significant.

We have previously shown in autoimmune disease models that absence of H2-O alters presentation of immunodominant epitopes by MHCII on antigen presenting cells (22). However, little has been reported about possible impacts of DO on epitope presentation during immune response against foreign antigens in infectious diseases. To address the question, we used inactivated H5N1 influenza vaccine as an immunogen and examined immune responses against HA protein by tracking the development of specific memory CD4 T cells and B cells in HLA-DR1 transgenic mice, with or without H2-O (methods and **Figure 1A**). We found similar levels of activated tetramer positive CD44+CD69+ H5N1-HA (259–274) CD4 T cells in both H5N1-Flu vaccine immunized DR1+H2-O WT and DR1+H2-O KO mice (**Figure S1**, **2**). However, we did notice a slightly higher percentage of CD44+DR1/H5N1-HA (259–274) tetramer positive CD4 T cells in DR1+H2-O WT mice than DR1+H2-O KO mice 4 months post immunization, but the differences were not statistically significant (**Figures 1B, C**). However, upon utilizing two long-lived memory CD4 T cell markers, CD99 and CD44 (41), we observed a significantly larger numbers of CD44+CD99+ CD4 memory T cells in DR1+H2-O WT mice than DR1+H2-O KO mice (**Figures 1D, E** & **Figure S3**). In addition, we detected fewer cells in both CD80+PD-L2+ and CD80-PD-L2+ memory B cell subsets (42) in the immunized DR1+H2-O KO mice (**Figures 1F, G** & **Figure S3**).

**Figure 2:**
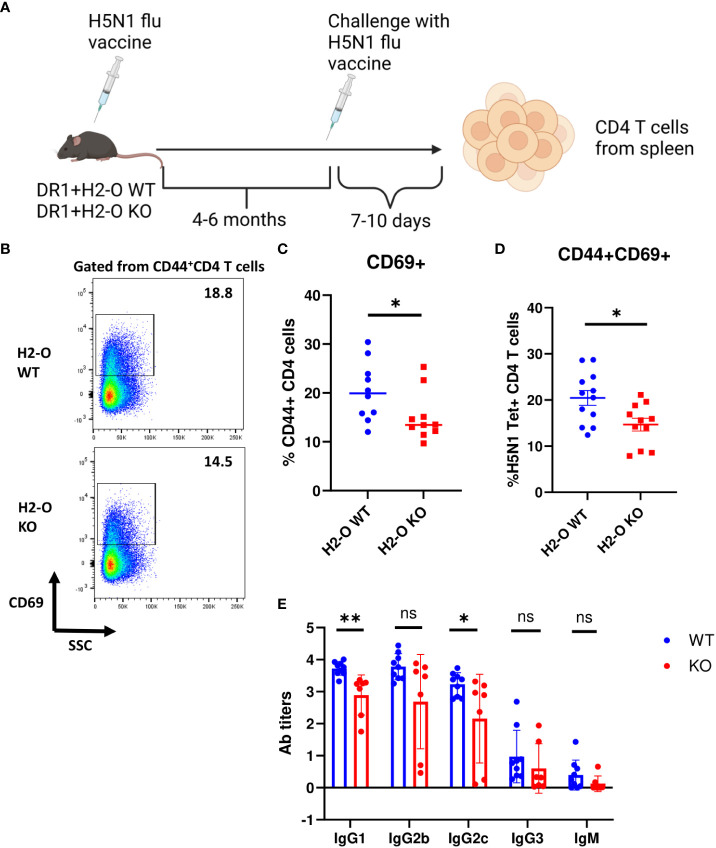
The function of memory cells was impaired in the flu vaccine immunized DR1+H2-O KO mice. **(A)** Schematic experimental design for testing memory response against flu. 6-8 weeks old DR1+H2-O WT mice and DR1+H2-O KO mice were intraperitoneally immunized with 9µg of inactivated H5N1 Influenza vaccine mixed with 50µg of CpG and challenged intraperitoneally with same dose of H5N1 Influenza vaccine in CpG 6 months post 1^st^ immunization. The mice were then sacrificed on day 6 post 2^nd^ immunization for spleens. The spleen cells were stained with CD3, CD4, CD44, CD69 antibodies and DR1/H5N1-HA (259–274) Tetramer (T cell panel) for flow cytometry. Each experiment including 4 mice per group was independently repeated 4 times. **(B)** Representative pseudocolor plots of CD69+ cells gated from CD44+ CD4 T cell population in spleen of immunized DR1+H2-O WT (Top) and DR1+H2-O KO (bottom) mice after *in vivo* challenge. **(C)** Percentages of CD69+ CD4 T cells in CD44+ CD4 T cell population from *in vivo* challenged immunized DR1+H2-O WT (Blue) and DR1+H2-O KO (Red) mice, P-value=0.0479 (two-tailed unpaired T-test). **(D)** Percentages of CD44+CD69+ CD4 T cells in DR1/H5N1-HA(259-274) Tetramer positive CD4 T cell population from *in vivo* challenged immunized DR1+H2-O WT (Blue) and DR1+H2-O KO (Red) mice, P-value=0.013 (two-tailed unpaired T-test). **(E)** Sera from DR1+H2-O WT (Blue) and DR1+H2-O KO (Red) mouse 10 days post *in vivo* challenge were collected and diluted 1:200 for indirect ELISA against H5N1-HA protein. 1:2000 diluted HRP conjugated Goat anti-mouse IgG1, IgG2b, IgG2c, IgG3 and IgM was used to detect the antibody in the serum. P-value=0.0104 (two-tailed unpaired T-test). Each dot represents one individual mouse. Data is shown as mean ± SEM. *p<0.05, **p<0.01, ns, not significant

To test if the observed differences at the level of memory CD4 T cells and memory B cells in H5N1-Flu vaccine immunized DR1+H2-O WT and DR1+H2-O KO mice translated into differences in memory responses, we challenged the H5N1-Flu vaccine immunized DR1+H2-O WT and DR1+H2-O KO mice with the same vaccine 4-6 months after the initial immunization, and examined the activation of memory T cells as well as HA specific antibody production (Methods and **Figure 2**). Over year of studying longevity of CD4 memory T cells *in vivo*, we have noted that quiescent memory T cells do not display significant phenotypic changes 4 months through the longest time we have studied i.e., 11 months (33, 41). We found larger memory responses in DR1+H2-O WT in comparison to DR1+H2-O KO as detected by larger percentages of activated CD69+ in total CD4 T cells and in H5N1-HA tetramer positive CD4 T cells (**Figures 2B-D**). Also, higher HA specific IgG1 and IgG2 antibody titers (**Figure 2E**) supported more effective memory B cell development in H5N1-Flu vaccine immunized DR1+H2-O WT.

### 3.2 No differences were found in follicular helper CD4 T cells or GC B cells in immune response between immunized H2-O WT and KO mice

**Figure 3:**
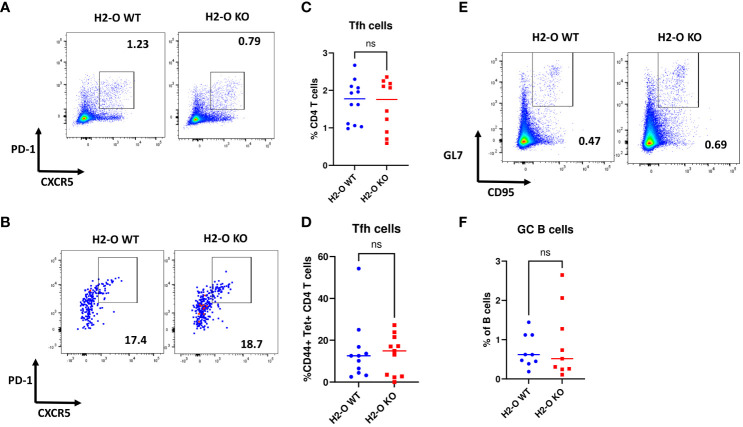
No differences were found in the percentages of germinal center B cells and follicular T help cells in immune responses between flu immunized DR1+H2-O WT and DR1+H2-O KO mice. 6-8 weeks old DR1+H2-O WT mice and DR1+H2-O KO mice were intraperitoneally immunized with 9µg of inactivated H5N1 Influenza vaccine mixed with 50µg of CpG and challenged intraperitoneally with the same dose of H5N1 Influenza vaccine in CpG, 4 months post 1^st^ immunization. The mice were then sacrificed on day 6 post 2^nd^ immunization for spleens. The spleen cells were divided and stained either with CD3, CD4, CD44, CD69, CXCR5, PD-1 antibodies and DR1/H5N1-HA(259-274) Tetramer (T cell panel) or B220, CD19, GL7, CD95 antibodies (B cell panel) for flow cytometry. Each experiment including 4 mice per group was independently repeated 3 times. **(A)** Representative pseudocolor plots of CXCR5+PD-1+ Tfh cells in total CD4 T cells from immunized DR1+H2-O WT (Left) and DR1+H2-O KO mice (Right). **(B)** Representative pseudocolor plots of CXCR5+PD-1+ Tfh cells in CD44+ H5N1 tetramer positive CD4 T cells from immunized DR1+H2-O WT (Left) and DR1+H2-O KO mice (Right). **(C)** Percentages of CXCR5+PD-1+ CD4 T cells gated from total CD4 T cell population in DR1+H2-O WT (Blue) and DR1+H2-O KO mice (Red). Each dot represents one individual mouse. P-value=0.6525(two-tail unpaired T-test). **(D)** Percentages of CXCR5+PD-1+ CD4 T cells gated from CD44+ H5N1 tetramer positive CD4 T cell population in DR1+H2-O WT (Blue) and DR1+H2-O KO mice (Red). Each dot represents one individual mouse. P-value=0.7536(two-tail unpaired T-test). **(E)** Representative pseudocolor plots of CD95+GL7hi B cells immunized DR1+H2-O WT (Left) and DR1+H2-O KO mice (Right). **(F)** Percentages of CD95+ GL7hi B cells gated from total CD19+ B220+ B cell population in DR1+H2-O WT (Blue) and DR1+H2-O KO mice (Red). Each dot represents one individual mouse. P-value=0.5718(two-tail unpaired T-test). Data is shown as mean ± SEM. ns, not significant.

Previous studies have indicated that absence of thymic H2-O alters CD4 T cells development leading to altered peripheral CD4 T cell repertoires between H2-O WT and H2-O KO mice (22). To investigate if altered CD4 T cell repertoires contribute to the faulty memory development observed in H2-O KO mice, we examined CXCR5+PD-1+follicular helper T cells (Tfh) (43) in H5N1-Flu vaccine challenged DR1+H2-O WT and DR1+H2-O KO mice (**Figures 1**, **2**). As shown in **Figures 3A-D**, no significant differences were found in the percentages of Tfh cells in total CD4 T cells or H5N1 tetramer positive CD4 T cells in memory response between H5N1-Flu vaccine immunized DR1+H2-O WT and DR1+H2-O KO mice. Moreover, there were no differences in the percentages of CD95+GL7hi GC B cells (44, 45) between DR1+H2-O WT and DR1+H2-O KO mice (**Figure S4**, **Figures 3E, F**), indicating that the GC reaction is probably not the source of faulty memory development observed in H5N1-Flu vaccine immunized DR1+H2-O KO mice. Additionally, we found no significant differences in the expression levels of costimulatory ligands on the surfaces of B cells and CD4 T cells in the H5N1-Flu vaccine immunized DR1+H2-O WT and DR1+H2-O KO mice (**Figure S5A**), except for CD86, which appeared to be significantly higher on B cells from both immunized and naive DR1+H2-O KO mice (**Figure S5B**).

### 3.3 OVA immunization led to normal memory development in H2-O KO mice

**Figure 4:**
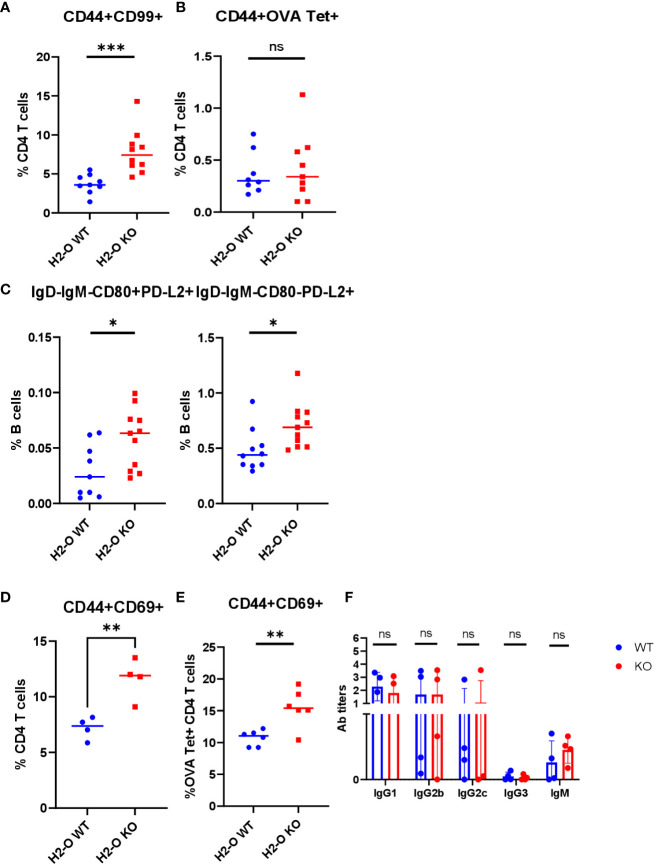
OVA immunization led to normal memory cell development in H2-O KO mice. 6-8 weeks old I-A^b^+H2-O WT and I-A^b^+H2-O KO mice were immunized intraperitoneally with 200µg of OVA protein and 50µg of CpG and sacrificed 5 months post immunization for spleen cells. The splenocytes were then divided for staining with either CD3, CD4, CD44 and CD99 antibodies and OVA tetramer (T cell panel) or B220, CD19, IgD, IgM, CD80 and PD-L2 antibodies (B cell panel) for flow cytometry. Each experiment including 4 mice per group was independently repeated 3 times. **(A)** Percentages of CD44+CD99+ CD4 T cells from spleens of immunized I-A^b^+H2-O WT mice (blue) and I-A^b^+H2-O KO mice (red). P-value=0.0009 for CD44+CD99+ memory CD4 T cell subsets. **(B)** Percentages of CD44+ OVA tetramer+CD4 T cells from spleens of immunized I-A^b^+H2-O WT mice (blue) and I-A^b^+H2-O KO mice (red). P-value=0.7031 **(C)** Percentages of IgD-IgM-CD80+PD-L2+ (Left panel) and IgD-IgM-CD80-PD-L2+ (Right panel) memory B cell subsets in total CD19+B220+ B cells from immunized I-A^b^+H2-O WT mice (Blue) and I-A^b^+H2-O KO mice (Red). The p-value for IgD-IgM-CD80+PD-L2+ cell subset between immunized I-A^b^+H2-O WT and I-A^b^+H2-O KO mice is 0.0215 (two-tailed unpaired T-test), the p-value for IgD-IgM-CD80-PD-L2+ cell subset between immunized I-A^b^+H2-O WT and I-A^b^+H2-O KO mice is 0.0189. **(D)** 6-8 week old I-A^b^+H2-O WT and I-A^b^+H2-O KO mice were intraperitoneally immunized with 200µg of OVA protein and 50µg of CpG and challenged intraperitoneally with same dose of OVA protein in CpG 6 months post 1^st^ immunization. The mice were then sacrificed on day 6 post 2^nd^ immunization for spleens. The spleen cells were stained with CD3, CD4, CD44, CD69 antibodies and I-Ab/OVA (323-339) Tetramer for flow cytometry. Percentages of CD44+CD69+ CD4 T cells in CD44+ CD4 T cell population from *in vivo* challenged immunized I-A^b^+H2-O WT mice (Blue) and I-A^b^+H2-O KO mice (Red), P-value=0.0055 (two-tailed unpaired T-test). **(E)** Percentages of CD44+CD69+ CD4 T cells in I-Ab/OVA(327-339) Tetramer positive CD4 T cell population from *in vivo* challenged immunized I-A^b^+H2-O WT mice (Blue) and I-A^b^+H2-O KO mice (Red) in **(D)**, P-value=0.0045 (two-tailed unpaired T-test). **(F)** Sera from I-A^b^+H2-O WT mice (Blue) and I-A^b^+H2-O KO mice (Red) 10 days post *in vivo* challenge in **(D)** was collected and 1:200 diluted for indirect ELISA against OVA protein. 1:2000 diluted HRP conjugated Goat anti-mouse IgG1, IgG2b, IgG2c, IgG3 and IgM was used to detect the antibody in the serum. The statistics were performed with two-tailed unpaired T-test. Data is shown as mean ± SEM. *p<0.05, **p<0.01, ***p<0.001, ns, not significant.

As described earlier, the H5N1-HA immunodominant epitope (HA259-274) is DM-resistant (21, 38), the binding of which to DR1 molecule would increase in the presence of DO (21), and be reduced in the absence of H2-O. Hence, a dampened immune response and faulty memory development would be expected. To test this hypothesis *in vivo*, we evaluated memory development against the well-characterized chicken OVA antigen. Advantageously, the immunodominant epitope of OVA (OVA326-339) peptide has recently been reported to be DM-sensitive (39), therefore absence of H2-O is not expected to impact OVA binding to I-A^b^ molecules in the same way as HA binding to DR1 molecules. As shown in **Figure 4**, interestingly, more memory CD4 T cells and B cells developed from OVA immunized H2-O KO mice five months post immunization. The higher numbers of memory CD4 T cells in the KO mice were accompanied with higher numbers of activated CD4 T cells after challenging the immunized mice with OVA 6 months post initial immunization (**Figures 4A-D**). Although the levels of antibody in the serum of challenged mice between WT and KO showed no significant differences, the KO mice did seem to have slightly more IgG2c than WT mice (**Figure 4F**).

### 3.4 Flu vaccine specific memory CD4 T cells do not become quiescent in H2-O KO mice, whereas the OVA memory CD4 T cells do

**Figure 5:**
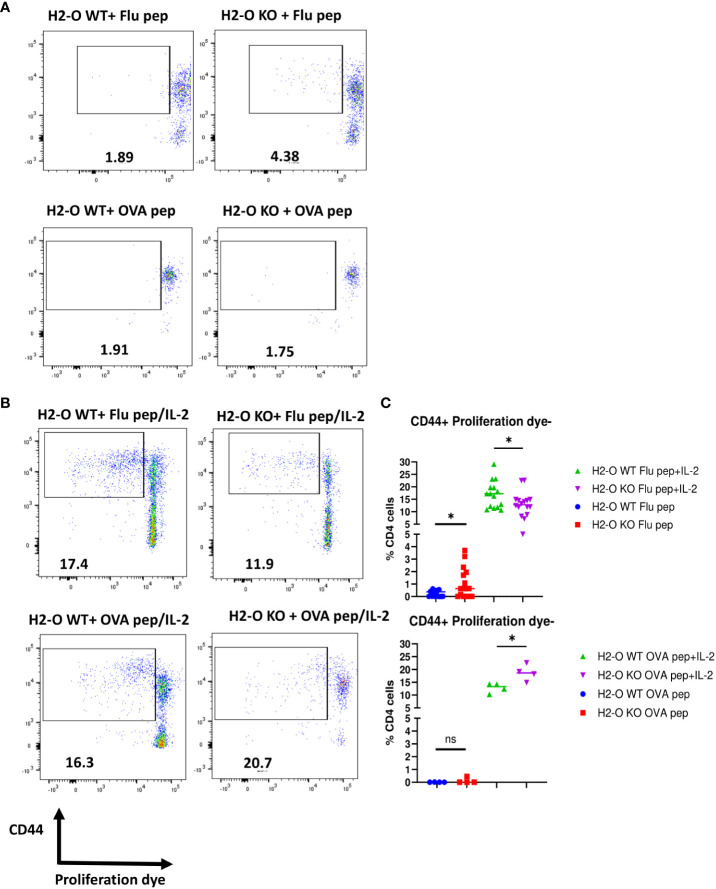
CD4 memory T cells from flu immunized DR1+H2-O KO mice were not quiescent. Flu vaccine immunized DR1+H2-O WT and DR1+H2-O KO mice (top panel) or OVA protein immunized I-A^b^+H2-O WT and I-A^b^+H2-O KO mice (bottom panel) were sacrificed 4-6 months post immunization for spleen cells. The splenocytes from each immunized mouse were labeled with either CFSE or Cell Trace Violet (CTV) proliferation dye and cultured *in vitro* with complete RPMI medium supplemented with 10% FBS for 6 days with the presence or absence of 1µM of H5N1-HA(259-274) peptide or OVA (326-339) peptide and 33cu of recombinant human IL-2. The cells were harvested on day 6 and stained with CD3, CD4, CD44 antibodies for flow cytometry. Each experiment including 4 mice per group was independently repeated 4 times. **(A)** Representative pseudocolor plots of CD44+proliferating DR1+H2-O WT (left) or DR1+H2-O KO (right) CD4 T cells with H5N1-HA(259-274) peptide only condition (top panel) and I-A^b^+H2-O WT (left) or I-A^b^+H2-O KO (right) CD4 T cells with OVA (326-339) peptide only condition (bottom panel). **(B)** Representative pseudocolor plots of CD44+proliferating DR1+H2-O WT (left) or DR1+H2-O KO (right) CD4 T cells with H5N1-HA (259-274) peptide/IL-2 condition (top panel) and I-A^b^+H2-O WT (left) or I-A^b^+H2-O KO (right) CD4 T cells with OVA (326-339) peptide/IL-2 condition (bottom panel). **(C)** (Top) Percentages of CD44^+^proliferation dye negative DR1+H2-O WT or DR1+H2-O KO CD4 T cells with either H5N1-HA(259-274) peptide only (Blue and Red) or H5N1-HA(259-274) peptide and recombinant human IL-2 together (Green and Purple). P-value=0.0418 for peptide and IL-2 condition, P-value=0.0185 for peptide only condition (two-tailed unpaired T-test). (Bottom) Percentages of CD44+proliferation dye negative I-A^b^+H2-O WT or I-A^b^+H2-O KO CD4 T cells with either OVA (326-339) peptide only (Blue and Red) or OVA (326-339) peptide and recombinant human IL-2 together (Green and Purple). P-value=0.0185 for peptide and IL-2 condition (two-tailed unpaired T-test). Each dot represents cells from one individual mouse. Data is shown as mean ± SEM. *p<0.05, ns, not significant.

Differences in the density of MHCII/peptide complexes presented on B cells has been well documented to be linked with proper memory CD4 T cell development characterized by quiescence, important for the longevity of memory CD4 T cells (29, 31, 33, 41). To examine whether the loss of H2-O could affect development of quiescent CD4 memory T cells, hence faulty memory development observed after H5N1-Flu vaccine immunization, we stimulated CD4 memory T cells from either Flu or OVA immunized H2-O WT and H2-O KO mice with peptide only, or peptide and IL-2 *in vitro* and tracked their proliferation. As shown in **Figure 5A**, stimulation with only the HA (259–274) peptide induced 3-fold more proliferation in CD4 memory T cells from H5N1-Flu vaccine immunized KO mice as compared to CD4 T cells from H5N1-Flu vaccine immunized WT mice, indicating that H2-O KO CD4 memory T cells were not quiescent. In contrast, both WT and KO CD4 memory T cells from OVA immunized mice remained unresponsive to OVA peptide stimulation alone, indicative of their quiescence. Interestingly, CD4 memory T cells from H5N1-Flu vaccine immunized WT mice exhibited significantly higher proliferation to an *in vitro* challenge to HA peptide and IL-2 supporting the *in vivo* challenge data shown in **Figure 2** (**Figure S6**, **Figures 5B, C**). The opposite trend was found for CD4 memory T cells from OVA immunized H2-O KO mice, where OVA peptide and IL-2 stimulation drove significantly more proliferation in H2-O KO memory CD4 T cells.

**Figure 6:**
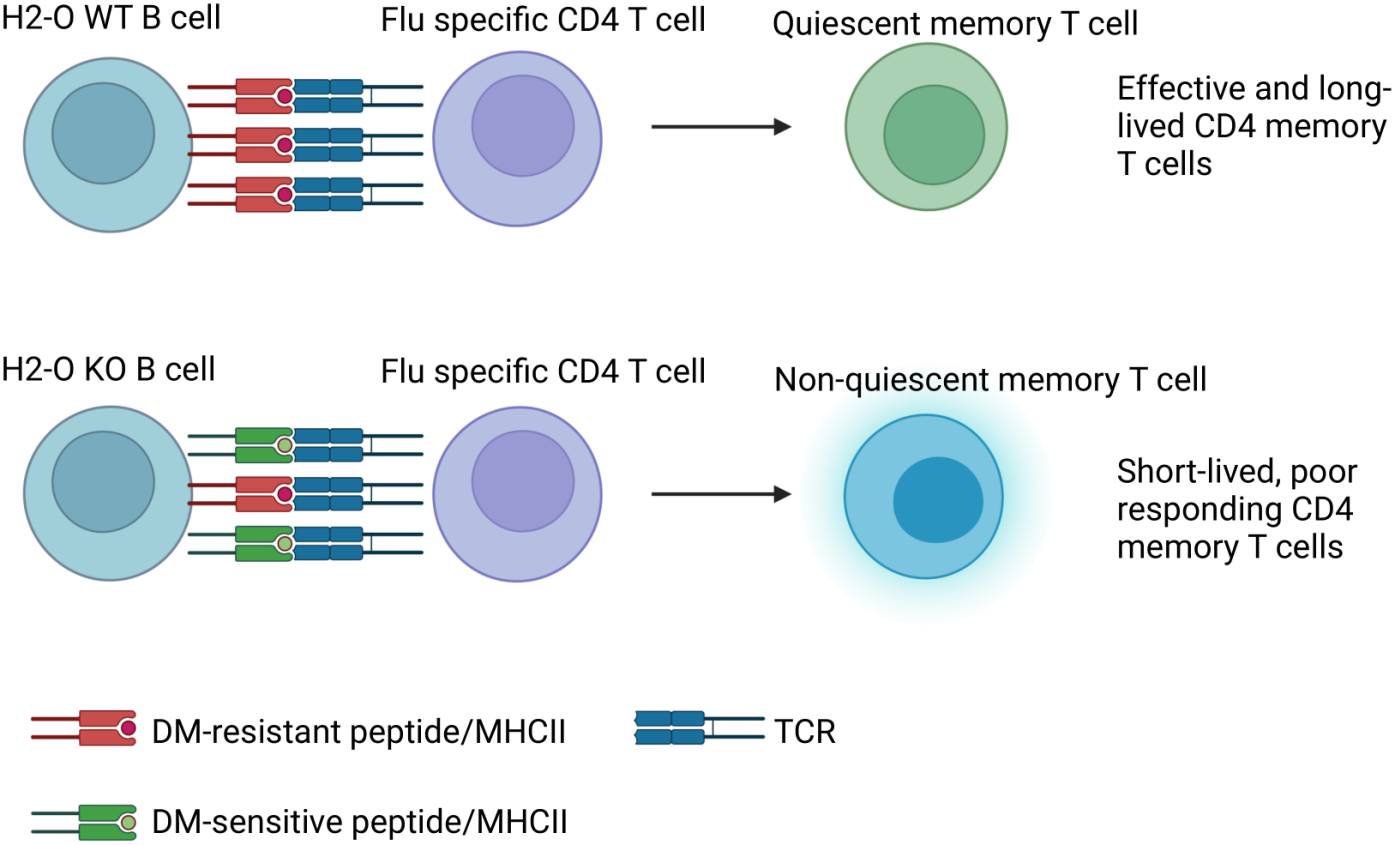
Model: In DR1+H2-O KO mice altered antigen presentation in GC B cells leads to failure in memory CD4 T cell development to H5N1-Flu vaccine immunization. Upon immunization with H5N1-Flu vaccine, B cells from H2-O WT mice present sufficient densities of DM-resistant immunodominant epitope/DR1 to Ag-experienced CD4 T cells in GC. On the contrary, B cells from H2-O KO are expected to present too low of antigen densities of DM-resistant/DR1 epitopes, insufficient for the induction of long-lived memory T cells.

In summary, absence of H2-O in mice negatively affects proper development of memory B cells and H5N1-HA (259–274) specific memory CD4 T cells, but not OVA (326–339) specific CD4 memory T cells (**Table 1**). This difference is likely due to differences in the densities of presented DM-resistant H5N1-HA epitope/DR1 complexes (**Figure 6**).

**Table 1:** Summary of memory cell development between flu vaccine and OVA immunized H2-O WT and H2-O KO mice.

	H2-O WT with Flu	H2-O KO with Flu	H2-O WT with OVA	H2-O KO with OVA
Proliferation of CD4 Tmem with *in vitro* stimulation of antigen + IL-2	++	+	+	++
Number of CD4 Tmem cells *in vivo*	++	+	+	++
Upregulation of CD69 expression in 2’ response	++	+	+	++
Number of Bmem cells *in vivo*	++	+	+	++
Antigen-specific IgG1 and IgG2 production in 2’response	++	+	+	+
Proliferation of CD4 Tmem with only peptide stimulation *in vitro*	NO	YES	NO	NO

## 4 Discussion

The proper development of memory T cells and B cells is crucial to development of efficient adaptive immune responses. Many groups have devoted efforts to understanding how immunological memory is established and maintained (46–49). In the past few years, much progress has been made towards understanding the metabolic requirement of CD8 T cells during memory development (3–5, 50, 51). Much less attention, however, has been given to the development of CD4 memory T cells. Our laboratory has previously made several important observations on how CD4 memory T cells gradually become quiescent and long-lived (29, 31, 33, 41). Using various quantities of agonist peptides, we have reported that *in vivo* presentation of low densities of agonist peptides drive CD4 memory T cells into a quiescent state characterized by lack of response to stimulation with specific peptides, but responsiveness to peptides plus IL-2 or other danger signals (33). Crucially, this quiescence state of CD4 memory T cells is mediated by B cells during the clearance of infection, when antigen reaches to lower than stimulatory levels (31). If the amount of antigen presented to memory CD4 T cells at the resolution of infection is too high or too low, this quiescence state would be disrupted and could lead to defects in the memory development (33).

Our model of how DO functions led to a hypothesis that presentation of lower densities of DM-resistant peptide-MHC II complexes by B cells in the absence of DO in periphery could disrupt the proper development of quiescent memory CD4 T cells. We tested this hypothesis using two different strains of H2-O knockout mice and two antigens carrying immunodominant epitopes that were either DM-resistant HA (259–274), or DM-sensitive, OVA (326–339). We hypothesized that absence of DO would lead to development of a non-quiescent state of memory CD4 T cells for HA epitope, but not for OVA epitope. In accord with our hypothesis, memory CD4 T cells from H5N1-flu immunized DR1+H2-O KO mice did not undergo quiescence, and importantly they did not respond to an *in vivo* H5N1 Flu vaccine challenge. Memory CD4 T cells from OVA immunized I-A^b^+H2-O KO mice, on the other hand behaved normally and developed resting quiescent memory CD4 T cells and responded well to *in vivo* recall. These findings further emphasize that DM-sensitivity of the antigenic epitopes together with DO determines differential development of memory CD4 T cell.

Quick secretion of high affinity, antigen specific antibodies by memory B cells and plasma cells is a highly important aspect of immunological memory (52–54). Interaction of CD4 T cells and B cells is the foundation for GC reaction and memory B cell development (55, 56). When we addressed contributions of H2-O to GC reaction, memory B cell development, and humoral response to antigens, we observed a decrease in the percentages of memory B cell subsets in the DR1+H2-O KO mice. Furthermore, *in vivo* challenge of H5N1 vaccine immunized mice led to lower titers of HA-specific IgG antibodies in DR1+H2-O KO mice in comparison to titers from DR1+H2-O WT mice. This reduction in the number of both memory B cells and antibody levels after H5N1-Flu vaccine immunization suggests that the absence of DO could have impaired the GC reaction. It is well established that expression of DM leads to the selection of immunodominant epitopes at higher densities (38, 57, 58), a lower expression of DM and DO in GC as reported (11, 59, 60) would allow for the presentation of a wider variety of peptides by B cells in GC. Based on our model, DO optimizes DM function, hence, downregulation of DO in B cells would lead to the presentation of lower densities of antigenic peptides for optimized selection of CD4 T cells with highest TCR affinity for those complexes.

While DO expression is downregulated in GC, one would wonder why we see a difference in memory CD4 development and longevity associated with the immune responses in H2-O KO versus H2-O WT mice. Naturally, despite a decreased expression of DM and DO in GC in the H2-O WT mice, in comparison to H2-O KO there is still some DO expressed. The faulty memory development in H2-O KO mice can also be affected by a reduction in trafficking of B cells to the GC. Higher levels of p/MHCII presentation by B cells is known to be a critical factor for entry to GC (61, 62), therefore, it is expected that in the presence of DO, recruitment of B cells to GC is more effective as compared to the H2-O KO. In one study, Draghi et al. have reported that H2-O KO B cells migrated to GC more effectively than H2-O WT B cells (60). Their observations are well in accord to our findings, as there are more self-reactive activated B cells are present in H2-O KO (12, 22), hence, they are more likely to traffic to GC to interact with self-reactive T cells.

Lack of H2-O lead to lower titers of IgG1 and IgG2 after HA immunization and recall signifying a role for DO in Ag-specific Ab production. These findings go against Denzin et al, who have suggested that lack of H2-O promotes production of broadly neutralizing antibodies against MMTV infection (25). One possible reason for this discrepancy could be that immunodominant epitopes of MMTV are DM-sensitive, whereas our H5N1-Flu immunodominant epitope is well characterized and is highly DM-resistant (21, 35, 38, 63). Differences between inactivated flu vaccine and a live virus could possibly be a reason (64). These observations encourage future investigations on how DO could impact antibody production against different viral antigens.

Overall speaking, our findings suggest that H2-O contributes to the GC reaction by modulating the levels of antigen presentation by B cells, which is important to ensure a proper immunological memory development after viral infections. The observation of a faulty memory response from CD4 T cells and B cells in the absence of H2-O further indicates that the role of H2-O in immune response against viral antigens has been underestimated. Critically, we emphasized that DM-sensitivity of antigenic epitopes are vital to effective vaccines.

## Data availability statement

The original contributions presented in the study are included in the article/**Supplementary Material**, further inquiries can be directed to the corresponding author/s.

## Ethics statement

The animal study was approved by Animal Care and Use Committee of the Johns Hopkins University School of Medicine. The study was conducted in accordance with the local legislation and institutional requirements.

## Author contributions

SS-N: Conceptualization, Funding acquisition, Methodology, Project administration, Resources, Supervision, Writing – review & editing. NS: Conceptualization, Data curation, Formal Analysis, Investigation, Methodology, Validation, Visualization, Writing – original draft, Writing – review & editing. RW: Conceptualization, Data curation, Formal Analysis, Investigation, Methodology, Resources, Validation, Visualization, Writing – original draft, Writing – review & editing.

## Supplementary material

Supplementary Figure 1 Gating strategy for tetramer staining and CD69 staining in primary response. 6-8 weeks old DR1+H2-O WT mice and DR1+H2-O KO mice were intraperitoneally immunized with 9µg of HA protein and 50µg of CpG and sacrificed for spleen cells 7-10 days post immunization. The splenocytes were then stained with CD3, CD4, CD44, CD69 antibodies and/or H5N1 HA(259-274) tetramer for flow cytometry. (Top) Representative pseudocolor plots of CD44+ H5N1 Tetramer+ CD4 T cells in immunized DR1+H2-O WT mice with or without tetramer staining. (Bottom) Representative pseudocolor plots of CD69+ CD4 T cells in immunized DR1+H2-O WT mice with or without CD69 antibody staining.

Supplementary Figure 2 No differences were observed in primary response between DR1+H2-O WT and DR1+H2-O KO after flu immunization. 6-8 weeks old DR1+H2-O WT mice and DR1+H2-O KO mice were intraperitoneally immunized with 9µg of HA protein and 50µg of CpG and sacrificed for spleen cells 7-10 days post immunization. The splenocytes were then stained with H5N1 HA(259-274) tetramer and CD3, CD4, CD44, CD69 antibodies for flow cytometry. The experiment has been repeated 3 times. **(A)** Representative pseudocolor plots of CD44+ CD69+ CD4 T cells in immunized DR1+H2-O WT mice (Left) and DR1+H2-O KO mice (Right). **(B)** Representative pseudocolor plots of CD44+H5N1 Tetramer+ CD4 T cells in immunized DR1+H2-O WT mice (Left) and DR1+H2-O KO mice (Right). **(C)** Percentages of CD69+ CD4 T cells in total CD44+CD4 T cells in immunized DR1+H2-O WT mice and DR1+H2-O KO mice (Left) and percentages of CD69+ cells in CD44+H5N1 Tetramer+ CD4 T cells in immunized DR1+H2-O WT mice and DR1+H2-O KO mice (Right). An unpaired t test has been performed for p values, p=0.5271(Left) and p=0.6942(Right). Data is shown as mean ± SEM. ns, not significant.

Supplementary Figure 3 Gating strategy for memory CD44+CD99+ CD4 T cell staining and CD80+PD-L2+ memory B cell staining. 6-8 weeks old DR1+H2-O WT mice and DR1+H2-O KO mice were intraperitoneally immunized with 9µg of inactivated H5N1 Influenza Vaccine and 50µg of CpG and sacrificed for spleen cells 4-6 months post immunization. The splenocytes were then divided for staining with either CD3, CD4, CD44 and CD99 antibodies (T cell panel) or B220, CD19, IgD, IgM, CD80 and PD-L2 antibodies (B cell panel) for flow cytometry. The experiment has been repeated 4 times. (Top) Representative pseudocolor plots of CD44+ CD99+ CD4 T cells in immunized DR1+H2-O WT mice with or without CD99 staining. (Bottom) Representative pseudocolor plots of CD80+PD-L2+ memory B cells in immunized DR1+H2-O WT mice with or without CD80 and PD-L2 antibody staining. CD4 memory T cells and memory B cells from OVA immunized H2-O WT and H2-O KO mice were gated using the same gating strategy.

Supplementary Figure 4 Gating strategy for CXCR5+PD-1+ CD4 Tfh cell staining and CD95+GL7hi GC B cell staining. 6-8 weeks old DR1+H2-O WT mice and DR1+H2-O KO mice were intraperitoneally immunized with 9µg of inactivated H5N1 Influenza vaccine mixed with 50µg of CpG and challenged intraperitoneally with same dose of H5N1 Influenza vaccine in CpG 4-6 months post 1^st^ immunization. The mice were then sacrificed on day 6 post 2^nd^ immunization for spleens. The spleen cells were divided and stained either with CD3, CD4, CD44, CD69, CXCR5, PD-1 antibodies and/or DR1/H5N1-HA(259-274) Tetramer (T cell panel) or B220, CD19, GL7, CD95 antibodies (B cell panel) for flow cytometry. (Top) Representative pseudocolor plots of CXCR5+PD-1+ CD4 Tfh cells or CD44+ H5N1 tetramer positive CXCR5+PD-1+ CD4 Tfh cells in immunized DR1+H2-O WT mice after *in vivo* challenge. No CXCR5 staining cells and no PD-1 staining cells are used as controls. (Bottom) Representative pseudocolor plots of CD95+GL7hi GC B cells in immunized DR1+H2-O WT mice after *in vivo* challenge. No CD95 staining cells and no GL7 staining cells are used as controls.

Supplementary Figure 5 No differences observed in surface costimulatory markers on CD4 T cells or B cells between H2-O WT and H2-O KO. **(A)** 6-8 week old DR1+H2-O WT mice and DR1+H2-O KO mice were intraperitoneally immunized with 9µg of inactivated H5N1 Influenza vaccine mixed with 50µg of CpG and challenged intraperitoneally with same dose of H5N1 Influenza vaccine in CpG 6 months post 1^st^ immunization. The mice were then sacrificed on day 10 post 2^nd^ immunization for spleens. The spleen cells were either stained with CD3, CD4, CD44, CD69, CD25, CD28, CD40L antibodies and DR1/H5N1-HA(259-274) Tetramer (T cell panel) or stained with B220, CD19, CD3, CD80, CD86 and CD40 antibodies (B cell panel) for flow cytometry. Normalized MFI levels of various cell surface costimulatory markers on CD4 T cells (CD28, CD40L) and B cells (CD80, CD86, CD40) in immunized DR1+H2-O WT mice (Blue) and DR1+H2-O KO mice (Red). To account for experimental variation, the average MFI level in H2-O WT samples was calculated. MFI levels in both H2-O WT and H2-O KO samples were then divided by the calculated H2-O WT average. An increased ratio indicates increased MFI levels. The statistics were performed with two-tailed unpaired T-test. P-value for CD86 between activated WT and KO B cells is 0.037 **(B)** Normalized MFI levels of CD86 on B cells of naive DR1+H2-O KO mice (Blue) and DR1+H2-O KO mice (Red). P-value for CD86 between naïve WT and KO B cells is 0.0119. Data is shown as mean ± SEM. *p<0.05, ns, not significant.

Supplementary Figure 6 Gating strategy for flu and OVA CD4 T cell proliferation after *in vitro* stimulation. Flu vaccine immunized DR1+H2-O WT and DR1+H2-O KO mice or OVA protein immunized I-A^b^+H2-O WT and I-A^b^+H2-O KO mice were sacrificed 4-6 months post immunization for spleen cells. The splenocytes from each immunized mouse were labeled with either CFSE or Cell Trace Violet (CTV) proliferation dye and cultured *in vitro* with complete RPMI medium supplemented with 10% FBS for 6 days with the presence or absence of 1µM of H5N1-HA(259-274) peptide or OVA (326-339) peptide and 33cu of recombinant human IL-2. The cells were harvested on day 6 and stained with CD3, CD4, CD44 antibodies for flow cytometry. (Top) Representative pseudocolor plots of CD44+proliferating DR1+H2-O WT CD4 T cells with H5N1-HA (259-274) peptide/IL-2 condition (upper panel) or media only condition as control (lower panel). CD44+ proliferating CD4 T cells from OVA immunized mice were gated using the same gating strategy. (Bottom)Representative histograms of CD44+proliferating DR1+H2-O WT or DR1+H2-O KO CD4 T cells with H5N1-HA(259-274) peptide only condition or H5N1-HA (259-274) peptide/IL-2 condition (upper panel). Modeling of CD4 T cell proliferation using FlowJo version 10 proliferation platform based on the histograms (lower panel). Division peaks of cells were color coded based on FlowJo prediction.
