# Supplementary figures and images for: Proper development of long-lived memory CD4 T cells requires HLA-DO function
Source: Front Immunol. 2023 Oct 16;14:1277609. doi: 10.3389/fimmu.2023.1277609 (PMC10613709; doi:10.3389/fimmu.2023.1277609)

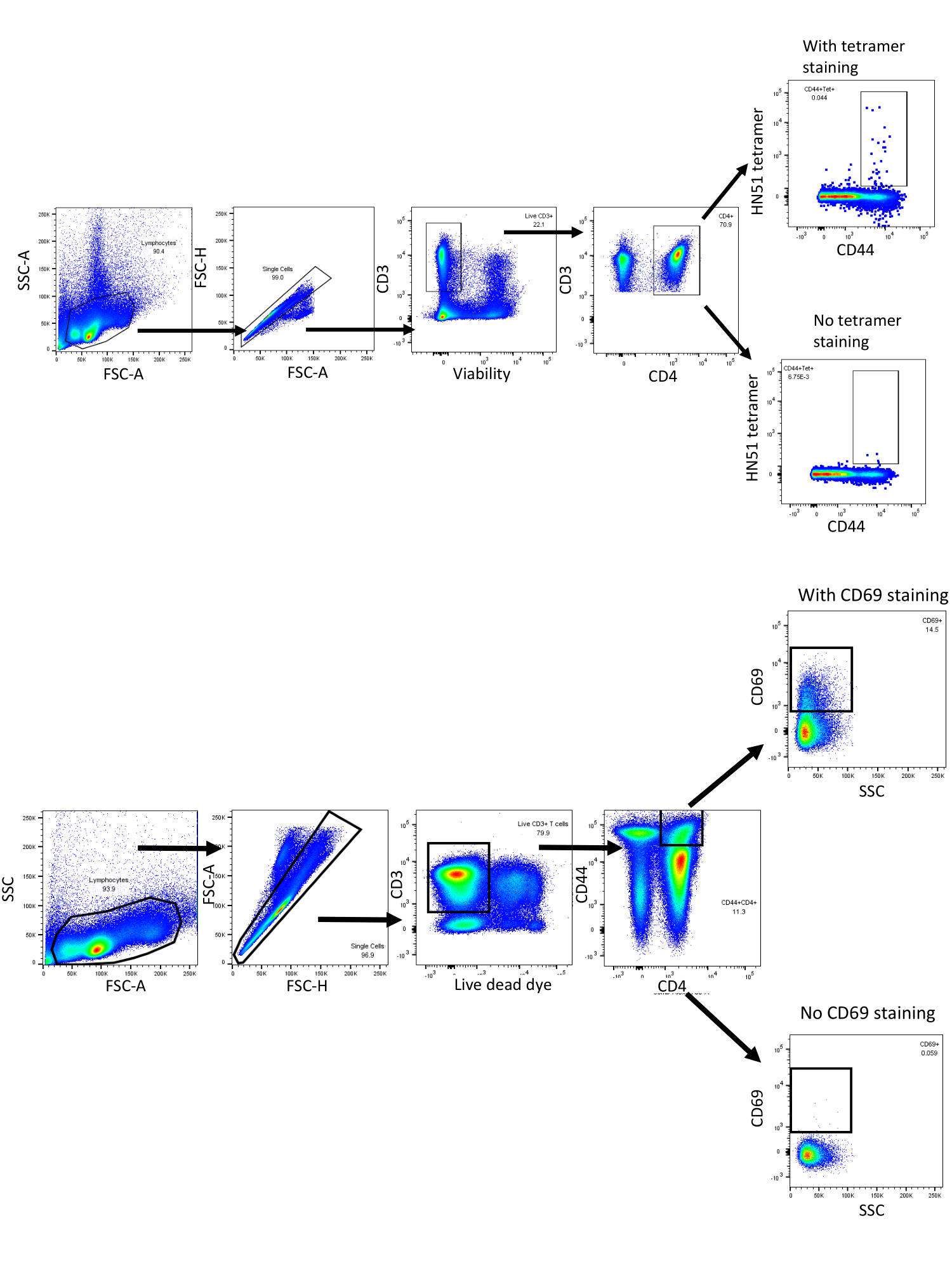

Supplement: Supplementary Figure 1 — Gating strategy for tetramer staining and CD69 staining in primary response. 6-8 weeks old DR1+H2-O WT mice and DR1+H2-O KO mice were intraperitoneally immunized with 9µg of HA protein and 50µg of CpG and sacrificed for spleen cells 7-10 days post immunization. The splenocytes were then stained with CD3, CD4, CD44, CD69 antibodies and/or H5N1 HA(259-274) tetramer for flow cytometry. (Top) Representative pseudocolor plots of CD44+ H5N1 Tetramer+ CD4 T cells in immunized DR1+H2-O WT mice with or without tetramer staining. (Bottom) Representative pseudocolor plots of CD69+ CD4 T cells in immunized DR1+H2-O WT mice with or without CD69 antibody staining. [file Image_1.tiff]

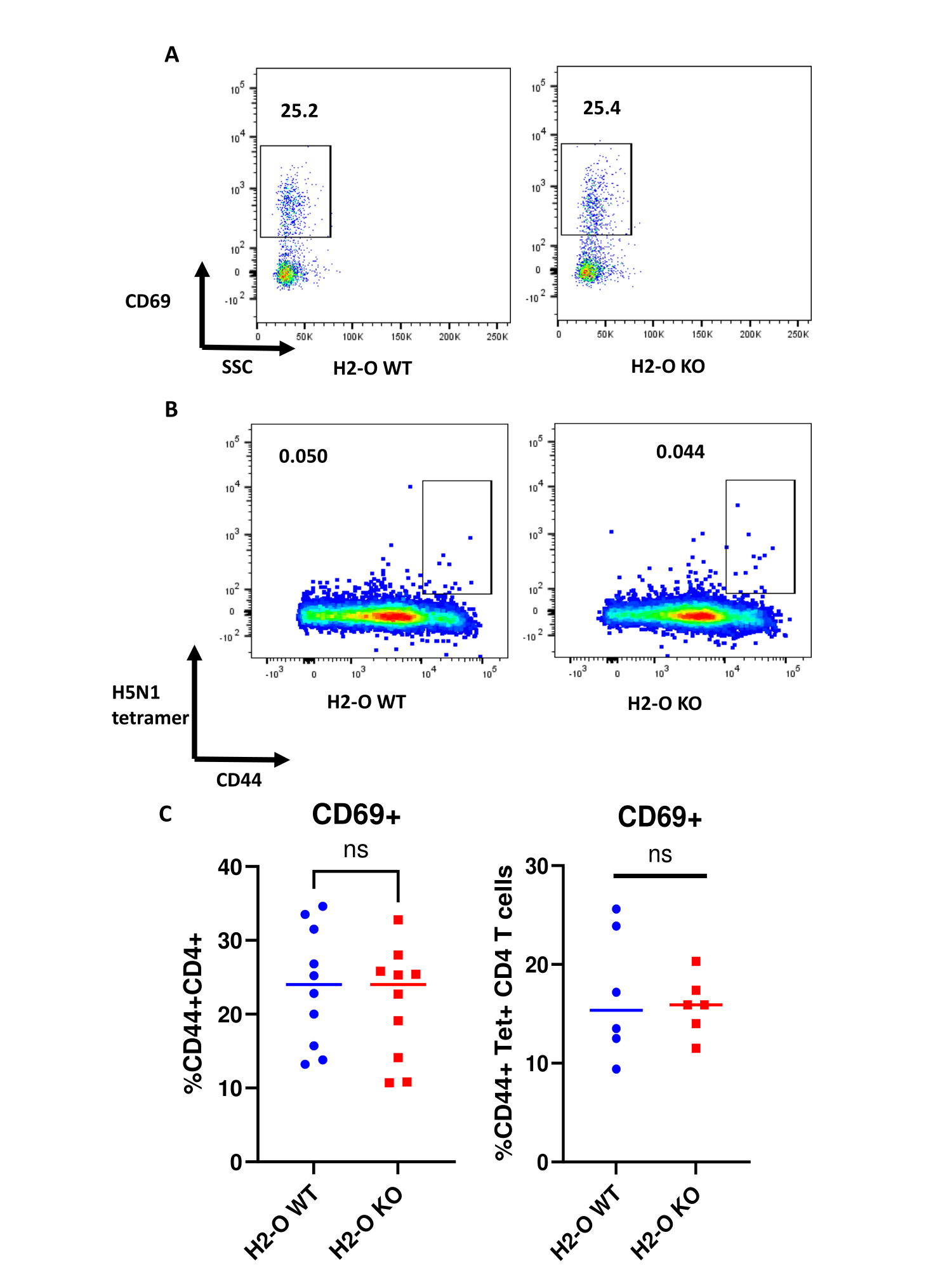

Supplement: Supplementary Figure 2 — No differences were observed in primary response between DR1+H2-O WT and DR1+H2-O KO after flu immunization. 6-8 weeks old DR1+H2-O WT mice and DR1+H2-O KO mice were intraperitoneally immunized with 9µg of HA protein and 50µg of CpG and sacrificed for spleen cells 7-10 days post immunization. The splenocytes were then stained with H5N1 HA(259-274) tetramer and CD3, CD4, CD44, CD69 antibodies for flow cytometry. The experiment has been repeated 3 times. (A) Representative pseudocolor plots of CD44+ CD69+ CD4 T cells in immunized DR1+H2-O WT mice (Left) and DR1+H2-O KO mice (Right). (B) Representative pseudocolor plots of CD44+H5N1 Tetramer+ CD4 T cells in immunized DR1+H2-O WT mice (Left) and DR1+H2-O KO mice (Right). (C) Percentages of CD69+ CD4 T cells in total CD44+CD4 T cells in immunized DR1+H2-O WT mice and DR1+H2-O KO mice (Left) and percentages of CD69+ cells in CD44+H5N1 Tetramer+ CD4 T cells in immunized DR1+H2-O WT mice and DR1+H2-O KO mice (Right). An unpaired t test has been performed for p values, p=0.5271(Left) and p=0.6942(Right). Data is shown as mean ± SEM. ns, not significant. [file Image_2.tiff]

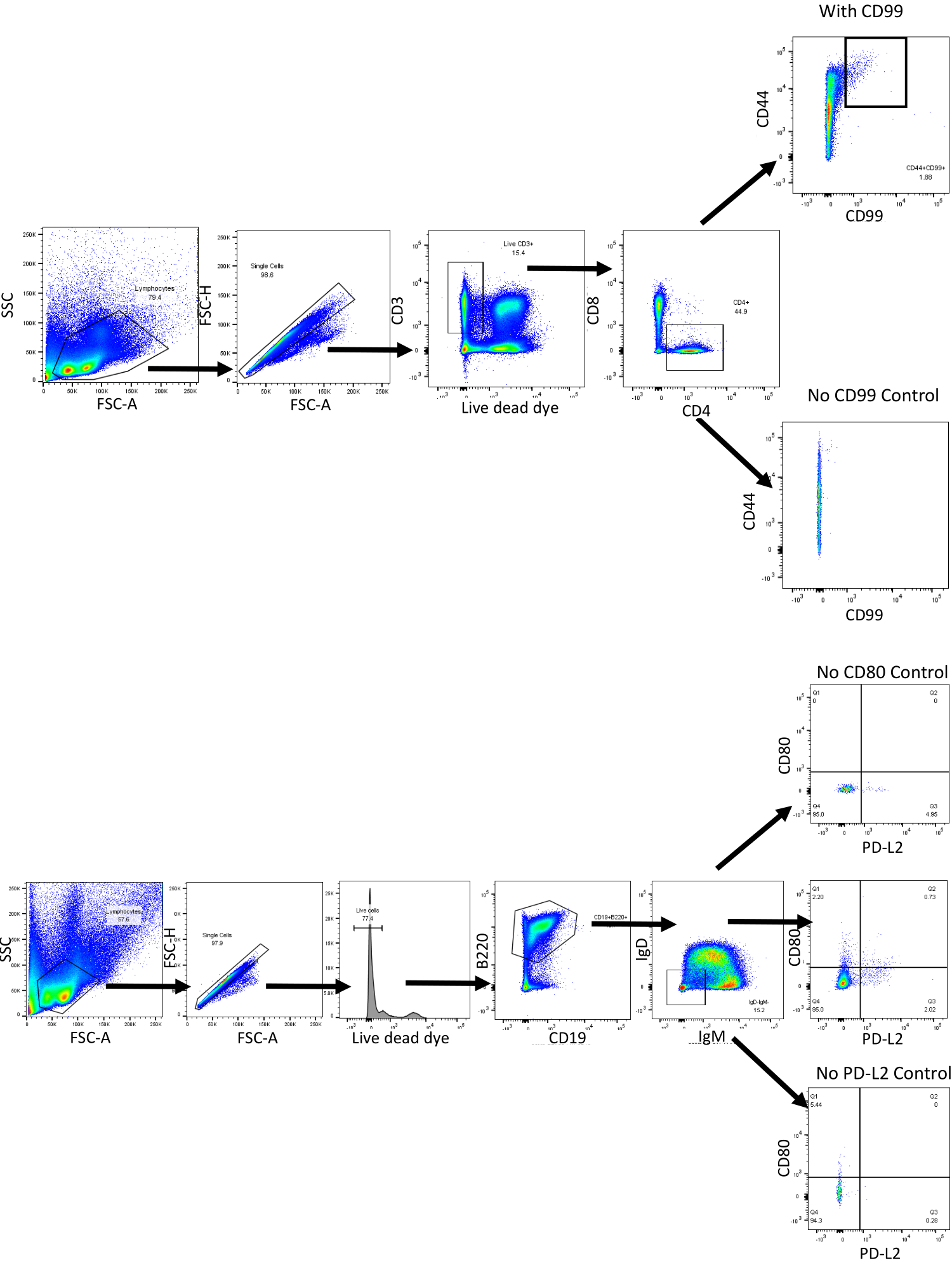

Supplement: Supplementary Figure 3 — Gating strategy for memory CD44+CD99+ CD4 T cell staining and CD80+PD-L2+ memory B cell staining. 6-8 weeks old DR1+H2-O WT mice and DR1+H2-O KO mice were intraperitoneally immunized with 9µg of inactivated H5N1 Influenza Vaccine and 50µg of CpG and sacrificed for spleen cells 4-6 months post immunization. The splenocytes were then divided for staining with either CD3, CD4, CD44 and CD99 antibodies (T cell panel) or B220, CD19, IgD, IgM, CD80 and PD-L2 antibodies (B cell panel) for flow cytometry. The experiment has been repeated 4 times. (Top) Representative pseudocolor plots of CD44+ CD99+ CD4 T cells in immunized DR1+H2-O WT mice with or without CD99 staining. (Bottom) Representative pseudocolor plots of CD80+PD-L2+ memory B cells in immunized DR1+H2-O WT mice with or without CD80 and PD-L2 antibody staining. CD4 memory T cells and memory B cells from OVA immunized H2-O WT and H2-O KO mice were gated using the same gating strategy. [file Image_3.tiff]

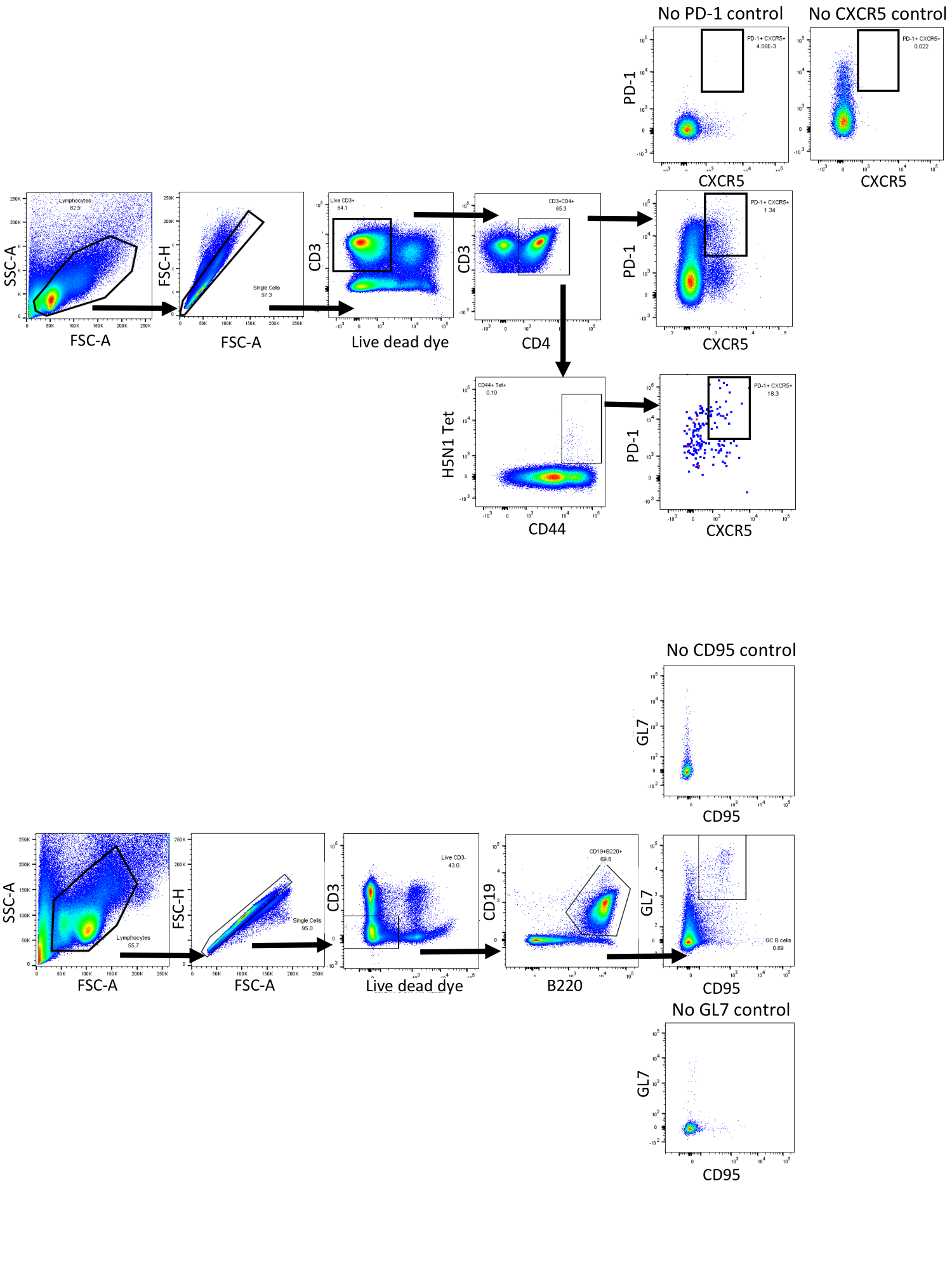

Supplement: Supplementary Figure 4 — Gating strategy for CXCR5+PD-1+ CD4 Tfh cell staining and CD95+GL7hi GC B cell staining. 6-8 weeks old DR1+H2-O WT mice and DR1+H2-O KO mice were intraperitoneally immunized with 9µg of inactivated H5N1 Influenza vaccine mixed with 50µg of CpG and challenged intraperitoneally with same dose of H5N1 Influenza vaccine in CpG 4-6 months post 1st immunization. The mice were then sacrificed on day 6 post 2nd immunization for spleens. The spleen cells were divided and stained either with CD3, CD4, CD44, CD69, CXCR5, PD-1 antibodies and/or DR1/H5N1-HA(259-274) Tetramer (T cell panel) or B220, CD19, GL7, CD95 antibodies (B cell panel) for flow cytometry. (Top) Representative pseudocolor plots of CXCR5+PD-1+ CD4 Tfh cells or CD44+ H5N1 tetramer positive CXCR5+PD-1+ CD4 Tfh cells in immunized DR1+H2-O WT mice after in vivo challenge. No CXCR5 staining cells and no PD-1 staining cells are used as controls. (Bottom) Representative pseudocolor plots of CD95+GL7hi GC B cells in immunized DR1+H2-O WT mice after in vivo challenge. No CD95 staining cells and no GL7 staining cells are used as controls. [file Image_4.tiff]

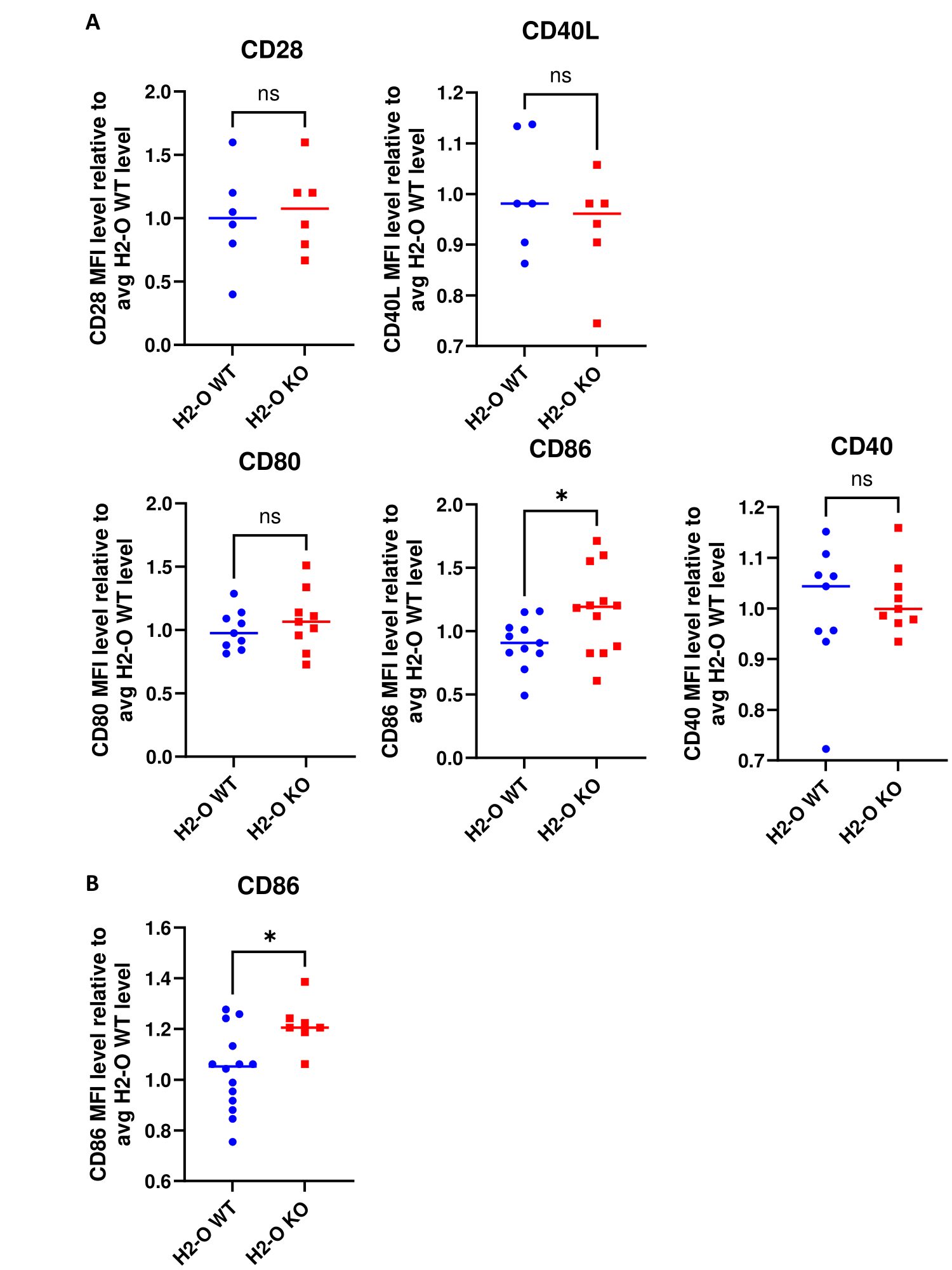

Supplement: Supplementary Figure 5 — No differences observed in surface costimulatory markers on CD4 T cells or B cells between H2-O WT and H2-O KO. (A) 6-8 week old DR1+H2-O WT mice and DR1+H2-O KO mice were intraperitoneally immunized with 9µg of inactivated H5N1 Influenza vaccine mixed with 50µg of CpG and challenged intraperitoneally with same dose of H5N1 Influenza vaccine in CpG 6 months post 1st immunization. The mice were then sacrificed on day 10 post 2nd immunization for spleens. The spleen cells were either stained with CD3, CD4, CD44, CD69, CD25, CD28, CD40L antibodies and DR1/H5N1-HA(259-274) Tetramer (T cell panel) or stained with B220, CD19, CD3, CD80, CD86 and CD40 antibodies (B cell panel) for flow cytometry. Normalized MFI levels of various cell surface costimulatory markers on CD4 T cells (CD28, CD40L) and B cells (CD80, CD86, CD40) in immunized DR1+H2-O WT mice (Blue) and DR1+H2-O KO mice (Red). To account for experimental variation, the average MFI level in H2-O WT samples was calculated. MFI levels in both H2-O WT and H2-O KO samples were then divided by the calculated H2-O WT average. An increased ratio indicates increased MFI levels. The statistics were performed with two-tailed unpaired T-test. P-value for CD86 between activated WT and KO B cells is 0.037 (B) Normalized MFI levels of CD86 on B cells of naive DR1+H2-O KO mice (Blue) and DR1+H2-O KO mice (Red). P-value for CD86 between naïve WT and KO B cells is 0.0119. Data is shown as mean ± SEM. *p<0.05, ns, not significant. [file Image_5.tiff]

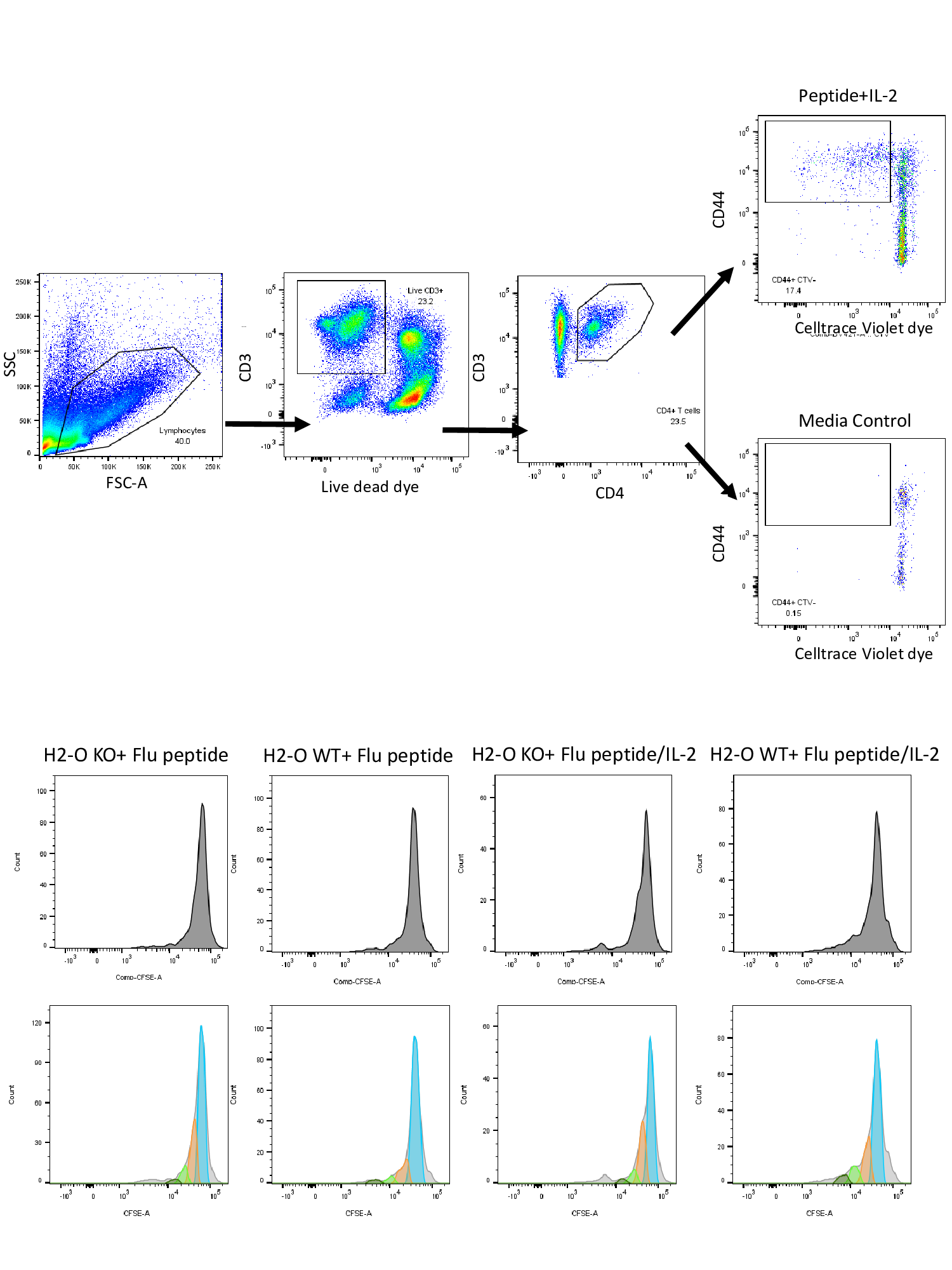

Supplement: Supplementary Figure 6 — Gating strategy for flu and OVA CD4 T cell proliferation after in vitro stimulation. Flu vaccine immunized DR1+H2-O WT and DR1+H2-O KO mice or OVA protein immunized I-Ab+H2-O WT and I-Ab+H2-O KO mice were sacrificed 4-6 months post immunization for spleen cells. The splenocytes from each immunized mouse were labeled with either CFSE or Cell Trace Violet (CTV) proliferation dye and cultured in vitro with complete RPMI medium supplemented with 10% FBS for 6 days with the presence or absence of 1µM of H5N1-HA(259-274) peptide or OVA (326-339) peptide and 33cu of recombinant human IL-2. The cells were harvested on day 6 and stained with CD3, CD4, CD44 antibodies for flow cytometry. (Top) Representative pseudocolor plots of CD44+proliferating DR1+H2-O WT CD4 T cells with H5N1-HA (259-274) peptide/IL-2 condition (upper panel) or media only condition as control (lower panel). CD44+ proliferating CD4 T cells from OVA immunized mice were gated using the same gating strategy. (Bottom)Representative histograms of CD44+proliferating DR1+H2-O WT or DR1+H2-O KO CD4 T cells with H5N1-HA(259-274) peptide only condition or H5N1-HA (259-274) peptide/IL-2 condition (upper panel). Modeling of CD4 T cell proliferation using FlowJo version 10 proliferation platform based on the histograms (lower panel). Division peaks of cells were color coded based on FlowJo prediction. [file Image_6.tiff]
